# Response of Merkel Cell Polyomavirus-Positive Merkel Cell Carcinoma Xenografts to a Survivin Inhibitor

**DOI:** 10.1371/journal.pone.0080543

**Published:** 2013-11-18

**Authors:** Lindsay R. Dresang, Anna Guastafierro, Reety Arora, Daniel Normolle, Yuan Chang, Patrick S. Moore

**Affiliations:** 1 Cancer Virology Program, University of Pittsburgh Cancer Institute, Pittsburgh, Pennsylvania, United States of America; 2 Institute for Stem Cell Biology and Regenerative Medicine, National Centre for Biological Sciences, GKVK Campus, Bangalore, India; 3 Biostatistics Facility, University of Pittsburgh Cancer Institute, Pittsburgh, Pennsylvania, United States of America; University of Southern California Keck School of Medicine, United States of America

## Abstract

Merkel cell carcinoma (MCC) is a neuroendocrine skin cancer associated with high mortality. Merkel cell polyomavirus (MCV), discovered in 2008, is associated with ~80% of MCC. The MCV large tumor (LT) oncoprotein upregulates the cellular oncoprotein survivin through its conserved retinoblastoma protein-binding motif. We confirm here that YM155, a survivin suppressor, is cytotoxic to MCV-positive MCC cells *in vitro* at nanomolar levels. Mouse survival was significantly improved for NOD-Scid-Gamma mice treated with YM155 in a dose and duration dependent manner for 3 of 4 MCV-positive MCC xenografts. One MCV-positive MCC xenograft (MS-1) failed to significantly respond to YM155, which corresponds with *in vitro* dose-response activity. Combination treatment of YM155 with other chemotherapeutics resulted in additive but not synergistic cell killing of MCC cell lines *in vitro*. These results suggest that survivin targeting is a promising therapeutic approach for most but not all MCV-positive MCCs.

## Introduction

Merkel cell carcinoma (MCC) is an aggressive non-melanoma skin cancer. Current therapies for MCC include surgical excision combined with radiation treatment [1,2,3,4]. However, the prognosis for patients with MCC is relatively poor, with a 2-year survival of 11% at stage IV (metastatic disease), a 5-year survival of 52% at stage III (disease with abnormal lymph nodes), and a 5-year survival of 67-81% at stages II-I (local disease) [2]; 25-30% of patients will already present with distal metastasis or lymph node abnormalities at the time of diagnosis [2,5]. Recent increases in MCC incidence [6,7,8,9] and association with immunocompromised conditions [7,10,11] prompted a search for an underlying viral cause. A novel human polyomavirus was discovered in MCC using digital transcriptome subtraction (DTS), a computationally-directed search for viral transcript sequences expressed in tumor tissues [12]. Merkel cell polyomavirus (MCV) has since been detected in ~80% of MCCs by multiple groups worldwide (reviewed by Kuwamoto [13]). MCV is found clonally integrated in MCC tumor cells, indicating that infection occurs prior to carcinogenesis [12,14,15,16].

Two viral proteins, MCV large tumor antigen (LT) and small tumor antigen (sT), contribute to MCC oncogenesis. Knockdown of both LT and sT results in cell death of MCV-positive MCC cell lines [17,18,19], as well as tumor regression in MCV-positive MCC xenografts [19]. Knockdown of sT alone results in growth arrest of MCC cell lines [19]. In all tumors examined to date, MCV LT is truncated by mutations that disrupt the LT helicase domain and render the virus replication incompetent [14,16]. The C-terminus of LT has recently been associated with anti-proliferative properties [20,21], which may provide a selective pressure to disrupt this region of LT during tumor initiation. Tumor-derived LT proteins, however, maintain a functional and conserved retinoblastoma protein (Rb) binding motif [12,14,15]. 

DTS analysis revealed that cellular genes are differentially expressed in MCV-positive MCCs, relative to MCV-negative MCCs. mRNAs for the cellular oncoprotein survivin were found to be seven-fold higher in virus positive, compared to virus negative MCC tumors [22]. This was not confirmed by a microarray analysis, suggesting either variability in tumors or technical differences in tumor dissection and mRNA detection [23]. Expression of both tumor-derived and wild-type MCV LT in BJ fibroblasts induces survivin expression unless the Rb-binding motif is mutated. Both transcript and protein levels of survivin decrease upon T antigen knockdown in several MCV-positive MCC cell lines, and knockdown of survivin results in cell death [22]. This has recently been confirmed by Xie et al [24]. While LT induction of survivin may be required for MCV-positive MCC cell survival, additional signaling pathways are also likely to be targeted by MCV LT [25]. 

A small molecule inhibitor of the survivin promoter, YM155 [26], was initially identified using a promoter luciferase reporter assay [26]. YM155 was able to diminish luciferase activity in a survivin promoter dependent context without cellular toxicity [26]. YM155 has since been shown to bind interleukin enhancer binding factor 3 (ILF3) [27], disrupting the ILF3/p54^nrb^ transcriptional complex at the survivin promoter, decreasing E2F1/2-mediated transcriptional activation of survivin [28]. YM155 antitumor activity has been demonstrated using a variety of cancer cell lines both *in vitro* and in mouse xenograft studies [29-35], and tested in phase I and II clinical trials for multiple malignancies [36-41]. Exploiting the apparent dependence of MCV-positive MCCs on survivin, YM155 was previously tested both *in vitro* and *in vivo* for MCC-specific cell killing with promising results [22].

We show here that YM155 is a potent inhibitor of MCC progression for most, but not all, MCV-positive MCC xenografts in NSG (non-obese diabetic, severe combined immunodeficient-gamma interleukin 2 receptor null) mice. While YM155 is toxic to MCV-positive MCC cells *in vitro*, the combination of YM155 with other common chemotherapeutic agents results in additive, but not synergistic, killing of MCV-positive MCC cells. Despite prolonged suppression of MCC growth in responsive mice, most mice were ultimately euthanized due to progressive MCC disease during YM155 treatment. Our results suggest that survivin targeting by small molecule inhibitors may be a promising approach to MCC therapy. 

## Materials and Methods

### Ethics Statement

All animal studies were performed with approval from the Animal Ethics Committee of the University of Pittsburgh (Institutional Animal Care and Use Committee Protocol #12020149). Tumor cell line injections, monitoring, and euthanasia were carried out under conditions to minimize suffering and in compliance with guidelines of the Hillman Cancer Center Animal Facility accredited by the Association for the Assessment and Accreditation for Laboratory Animal Care International.

### Cell Lines and Tissue Culture

The MCC cell lines MKL-1 [42,14], MS-1 [43], MKL-2 [44], and WaGa (gift of J. Becker [19]) were cultured in RPMI 1640 with 10% fetal calf serum, and primary human fibroblasts, BJ (American Type Culture Collection), were cultured in Dulbecco’s modified Eagle’s medium with 10% fetal calf serum, as described previously [22,43]. All cells were maintained at 37°C in humidified air containing 5% CO_2_. 

### NOD-Scid-Gamma Mice

The animals used for these studies are as described previously [22]. NSG female mice [45], strain #005557 (Jackson Laboratory), were received at 6-weeks of age and maintained in a specific, pathogen-free environment at the Hillman Cancer Center Mouse Facility, University of Pittsburgh, for at least one week prior to cell line injection. All animal studies were performed with approval from the Animal Ethics Committee of the University of Pittsburgh (Institutional Animal Care and Use Committee Protocol #12020149). 

### Xenografts and Treatment

**Figure 1:**
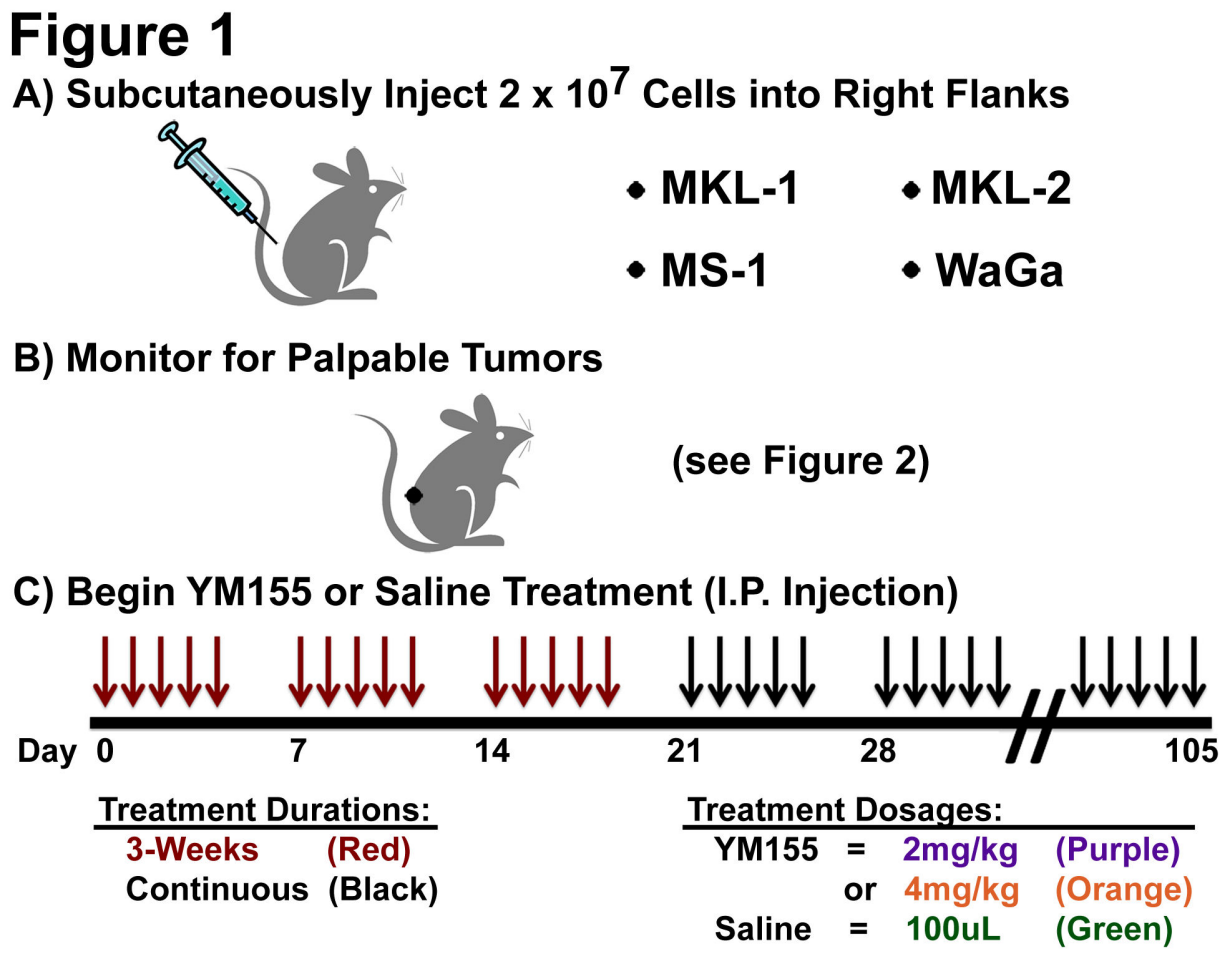
MCC mouse xenograft treatment groups and experimental outline. **A**) NSG mice were subcutaneously injected in the right flank with 2x10^7^ MCV-positive, MCC cells (MKL-1, MS-1, WaGa, or MKL-2). **B**) NSG mice were monitored for palpable tumors (~2mm x 2mm) to determine start of treatment. **C**) Mice with palpable tumors were randomly assigned to either saline treatment, YM155 treatment for 3-weeks at 2mg/kg, YM155 continuous treatment at 2mg/kg, or YM155 continuous treatment at 4mg/kg. Each week of treatment consisted of a single intraperitoneal injection per day for 5 days, followed by 2 days of rest.

MCC xenografts were generated as previously described [22]. MCC cell lines were optimally grown at >90% cell viability as determined by trypan blue dye exclusion. MCC cells were washed with phosphate-buffered saline (PBS) and resuspended at 2x10^7^cells per 100uL in PBS and injected into the right flanks of NSG mice. Tumor cell line injections were carried out under isoflurane anesthesia to minimize suffering. Treatment regimens began as individual animals developed palpable tumors (~2mm x 2mm), as outlined in Figure **1**. All treatments followed a five day on, two day off regimen of daily intraperitoneal (I.P.) injections. Three-week treatments ended on day 19 of I.P. injection. Continuous treatments were carried out until the animals reached the experimental endpoint. The experimental endpoint was evaluated by tumor burden, with at least one measurable diameter of 20mm, or by the presence of multiple signs of distress (>20% weight loss, behavioral changes, inactivity, or ruffled fur). Saline-treated mice were injected with a fixed volume of 100uL 0.9% Sodium Chloride USP Normal Saline (Nurse Assist) per injection. YM155 was administered at either 2mg/kg, 4mg/kg, or 6mg/kg, resuspended in 0.9% Sodium Chloride USP Normal Saline and filter sterilized. Tumor volumes were measured three times weekly and at the time of euthanization according to the following formula: width^2^ x length ÷ 2. Mouse weights were monitored at least once per week throughout the experiment. Observations, including weight measurements, were recorded daily on an individual mouse basis if signs of distress were observed.

### Statistical Analysis of Survival and Tumor Volume Data

Mixed-effects ANOVA was used for batch-adjusted times to 50% survival per cell line and treatment group, with 95% confidence intervals. Pairwise comparisons between treatments or between cell lines were estimated (with 95% confidence intervals) by linear contrasts on the estimated ANOVA parameters [46,47]. Between-batch variation was taken into account for all analyses. Tumor volumes were assessed for differential growth across treatment groups using an extension to the piecewise linear hierarchical Bayesian model [48] that accounts for batch effects. All analyses were performed using SAS (SAS Institute), R (R Development Core Team) and JAGS software [49]. Average tumor growth kinetics with 95% confidence intervals were estimated as described previously [22]. Briefly, a delay in tumor growth (or re-growth) is estimated by a hinge point, called nadir, where the volume at nadir (α) is expressed as a log_2_(volume) and the time at nadir (ρ) is expressed in days. An initial decrease in growth is estimated as β_1_, where log_2_(volume) = α+β_1_*(ρ-day). Final increase in tumor growth is estimated as β_2_, where log_2_(volume) = α+β_2_*(day-ρ). These four parameters are estimated for each animal and for each treatment and cell line. 

### Immunohistochemistry

Immunohistochemistry was performed as described previously [15]. Tumor and/or normal mouse tissue was cut to size for optimal formalin infusion (10% neutral-buffered solution; Sigma) for at least 24hrs prior to paraffin embedding. Paraffin embedding, preparation of unstained slides, and H&E processing was performed by Research Histology Services at the Thomas E. Starzl Transplantation Institute core facilities at the University of Pittsburgh. Unstained slides were baked at 60°C for 1hr under vacuum. Deparafinization continued with xylene treatment (2-3 incubations, 10min). Slides were gradually rehydrated moving from 100% ethanol (2 incubations, 10min), to 95% ethanol (2 incubations, 10min), to 80% ethanol with agitation, to 70% ethanol with agitation, and finally moving to deionized water. Slides were treated with 3% hydrogen peroxide to quench endogenous peroxidases, rinsed several times with deionized water, and then placed in 1mM EDTA pH8.0 for heat-induced epitope retrieval (125°C for 3min and 15s, followed by 90°C for 15s). After 45-60min of gradual cooling, slides were briefly rinsed several times with deionized water, rinsed with TBS (68mM NaCl, 10mM Tris pH7.5), treated with Protein Block (DAKO) for 5min, and then incubated with CM2B4 (15) primary antibody (diluted 0.5-1.5ug/mL) for 30min (PBS pH7.4, 1% BSA, 0.1% gelatin, 0.5% Triton-X-100, 0.05% sodium azide). Slides were washed 3 times with agitation in TBS. Secondary mouse-HRP antibody (Mouse Envision Polymer; DAKO) was incubated on the slides for 30min. Slides were again washed 3 times with agitation in TBS. Colorimetric detection with 3,3-diaminobenzidine and chromagen was quenched with deionized water. Slides were counter-stained with Mayer’s hemotoxylin, Lillie’s modification (DAKO), rinsed several times in tap water, blued in 1% lithium carbonate, rinsed several times in tap water, and then dehydrated through 95% ethanol (twice with agitation) to 100% ethanol (twice with agitation). Slides were incubated twice in xylene for 5min and coverslips were adhered using Permount (Fisher Scientific). 

### Chemotherapeutic Compounds

YM155 was purchased from Active Biochemicals Ltd. Docetaxel, carboplatin, etoposide, topotecan HCl, and bortezomib were provided by the NCI/DTP Open Chemical Repository (http://dtp.cancer.gov). 

### Dose-Response Studies

Dose-response studies were performed as previously described [22]. Briefly, 6000 cells were seeded per well in 384 well plates at a volume of 50uL and allowed to incubate overnight at 37°C in a 5% CO_2_ humidified chamber. A log range of drug concentrations from 10^-4^ to 10^-10^ was resuspended in culture medium (with or without a fixed amount of YM155) at 3X concentration and then added at a volume of 25uL. After 48 hours further incubation in a humidified chamber, cells were treated with 25uL CellTiter-Glo Luminescent reagent (Promega) and cell viability was measured as per manufacturer’s instructions. No-drug control wells served as normalization controls per cell line. Each concentration per cell line was plated in triplicate. Three or more biological replicates per cell line were tested with YM155 alone, two or more biological replicates were tested with all other single drugs, and combination studies were tested independently with 3nM YM155, with representative analysis at 3nM YM155 combination shown. Empty wells were used to separate different cell lines and treatment groups to reduce error from luminescent bleed-over. EC50 values were calculated from a four-parameter logistic equation fit to the surviving proportions of cells per dose. 

## Results

### MCV-Positive MCC Cell Lines Injected Subcutaneously in NSG Mice Have Variable Growth Rates

**Figure 2:**
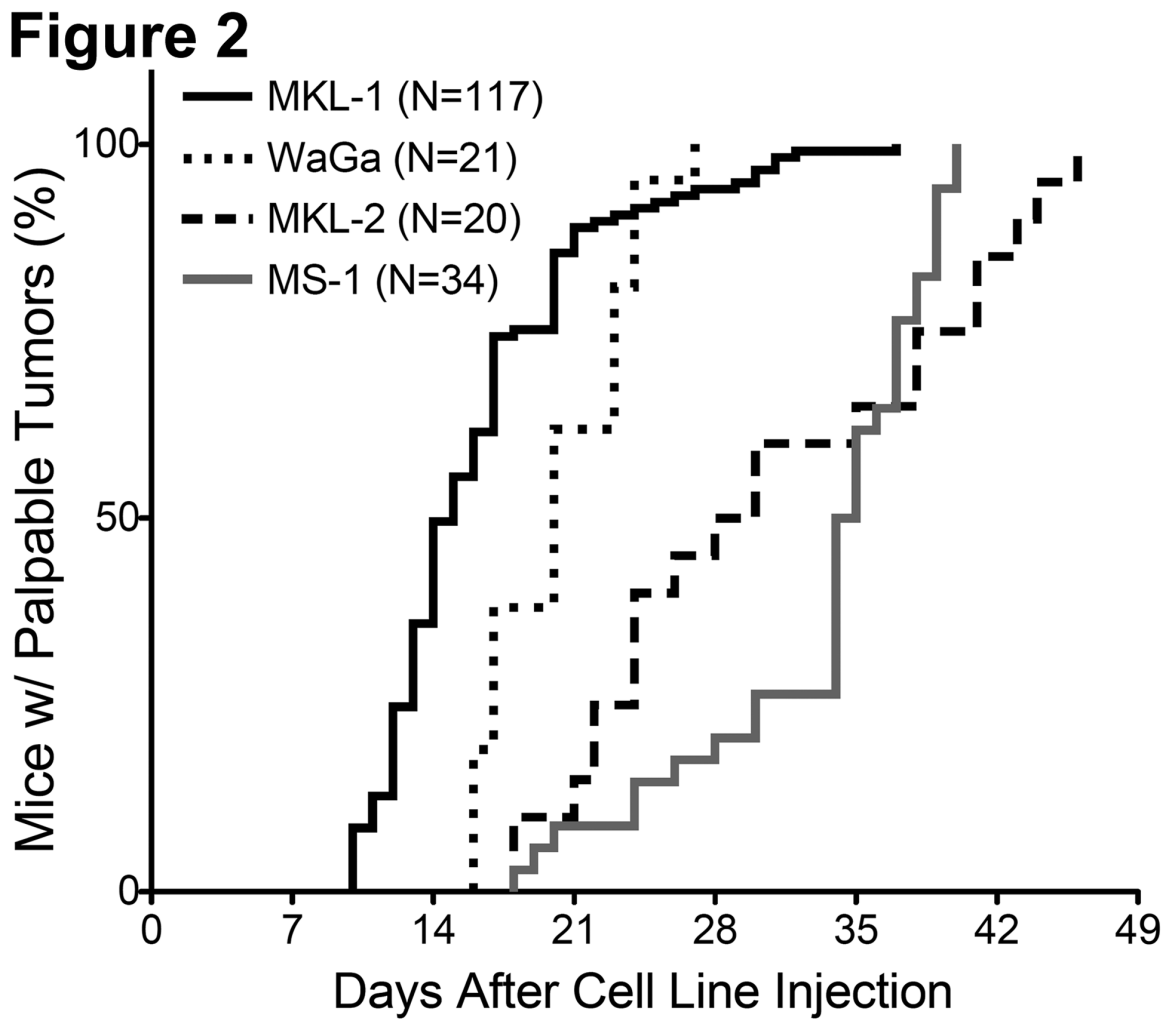
Time-to-Palpability. The length of time lapsed after initial cell line injection to detection of palpable tumors (~2mm x 2mm) is indicated for each of the four MCC cell lines tested (MKL-1, WaGa, MKL-2, and MS-1).

NSG mice were injected with MCV-positive MCC cell lines (Figure **1A**) and were monitored for tumor growth, weight (Figure S1 in File **S1**), and overall health. The length of time between cell line injection and detection of palpable tumors varied over a range for each cell line (Figure **2**). Overall, the time until 50% of mice had detectable, palpable tumors after cell line injection was shortest with MKL-1 xenografts, followed by WaGa, MKL-2, and finally MS-1. 

**Figure 3:**
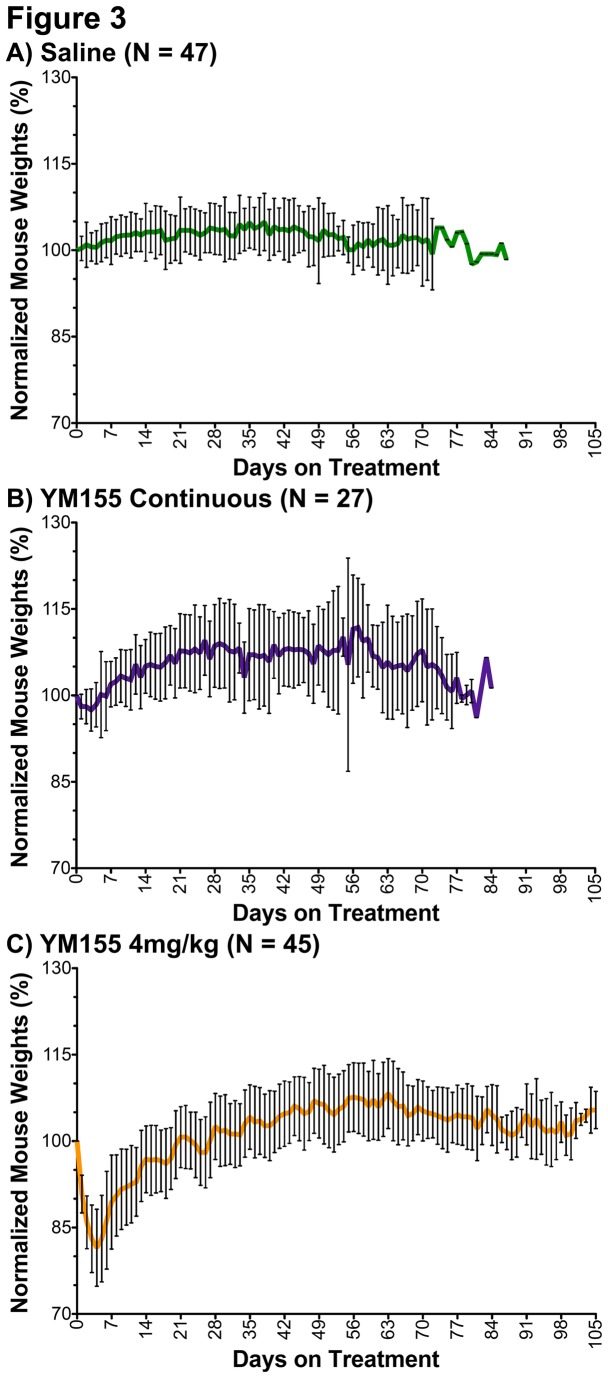
Mouse weights by treatment regimen. Average mouse weights with standard deviations are reported according to treatment regimen, where weights were normalized to day zero of treatment (100%): **A**) mouse weights on saline, continuous-treatment (green line); **B**) mouse weights on 2mg/kg YM155, continuous-treatment (purple line); and **C**) mouse weights on 4mg/kg YM155, continuous-treatment (orange line). Mouse weights were adjusted to remove the weight of tumors prior to normalization. Weights from mice with significant liver metastases were not included as metastatic-tumor weights could not be determined during the course of treatment.

Once palpable tumors were detected (Figure **1B**), NSG mice were intraperitoneally (I.P.) injected (once per day for five days, followed by two days of rest, Figure **1C**) with either saline treatment, 2mg/kg YM155 treatment for three weeks, or were continuously treated with YM155 (2mg/kg or 4mg/kg) until the tumor attained a diameter of 20mm or the mouse exhibited multiple signs of distress. YM155 at 6mg/kg was tested in two mice, but both mice had >20% weight loss and additional signs of distress (ruffled fur, inactivity, and behavioral changes) within the first week of treatment and were euthanized (as per Institutional Animal Care and Use Committee protocol #12020149). Mice receiving saline treatment or YM155 treatment at 2mg/kg do not lose weight (Figure **3A and 3B** **,** respectively) or show signs of distress, whereas mice receiving YM155 at 4mg/kg lose weight (Figure **3C**) and display minimal signs of distress (only ruffled fur, normal behavior). This toxicity dissipates after the first 1-2 weeks of treatment (Figure **3C**). Thus, 4mg/kg YM155 is the maximum tolerated dose in NSG mice when administered by single daily I.P. injection. 

Survival of mice with MCC xenografts is prolonged from the start of treatment by increasing YM155 duration of treatment, as well as by increasing the dosage of YM155, in a cell line dependent manner

**Figure 4:**
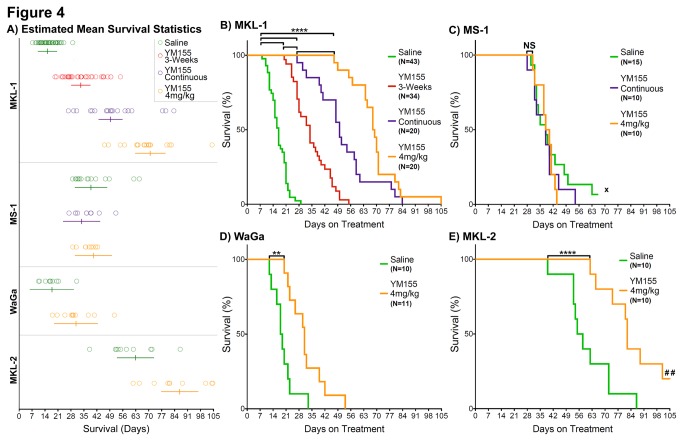
Kaplan-Meier curves of multiple MCC mouse xenograft models on different treatments. **A**) Estimated survival means and 95% confidence intervals are reported along compressed survival summaries per cell line and treatment arm, where open circles correspond survival of individual mice. **B**) Mice with MKL-1 xenografts exhibit significantly prolonged survival (****P < 0.0001) on any of the three YM155 treatment groups (3-weeks at 2mg/kg = red; continuous treatment at 2mg/kg = purple; continuous treatment at 4mg/kg = orange) relative to saline treatment (green). Increasing the duration of YM155 treatment from 3-weeks to continuous treatment at the 2mg/kg dose significantly prolongs survival (****P < 0.0001). Increasing the dose of YM155 from 2mg/kg to 4mg/kg on continuous treatment significantly prolongs survival (****P < 0.0001). **C**) Mice with MS-1 xenografts do not exhibit prolonged survival with YM155 continuous treatment (either at 2mg/kg or 4mg/kg) relative to saline treatment (NS = not significant). One mouse on saline treatment spontaneously regressed for over 5-weeks and was euthanized early (as indicated by **x**). **D**) Mice with WaGa xenografts exhibit significantly prolonged survival (**P = 0.0034) with continuous YM155 treatment at 4mg/kg relative to saline treatment. **E**) Mice with MKL-2 xenografts exhibit significantly prolonged survival (****P < 0.0001) with continuous YM155 treatment at 4mg/kg relative to saline treatment. Two mice did not reach the final 20mm tumor dimension by day 105 and were euthanized early (as indicated by **##**).

Estimated mean survival times with 95% confidence intervals are presented in Table S1 in File **S3** and Figure **4A** according to treatment group and cell line. Batch variations from independent replicates per treatment group and cell line were taken into account for the reported statistical analyses. Comparisons of estimated mean survival times across treatment groups or across cell lines are indicated in Table S2 in File **S3**. Figure **4B** shows a Kaplan-Meier survival curve for MKL-1 xenografts treated for a single 3-week course (2mg/kg YM155) or continuously until sacrifice (data from this figure include MKL-1 bearing mice treated in preliminary studies, published in [22]). Extending the duration of YM155 treatment prolongs survival (relative to saline or 3-week treatment, P<0.0001; Table S2 in File **S3**), which is prolonged further by doubling the YM155 dose to 4mg/kg (relative to all treatment arms, P<0.0001; Table S2 in File **S3**). 

We find EC50 values for YM155 *in vitro* range from 1.5nM to 12nM for different MCV-positive MCC cell lines (Table S3 in File **S3**), which are nearly identical to those previously described [22]. MKL-1 and MS-1 are at opposite ends of this range, respectively. MS-1 was tested in mice to assess the degree of response to YM155 *in vivo*. Mice were treated with either saline, 2mg/kg YM155 continuously, or 4mg/kg YM155 continuously as outlined in Figure **1**. In contrast to MKL-1, Figure **4C** and Table S2 in File **S3** show that MS-1 does not significantly respond to YM155 treatment *in vivo*, despite extended duration of treatment or increased dosage. This data is consistent with a lack of overall response to YM155 in MS-1 bearing mice, which was observed in our previous pilot comparison [22] (mice were not included here because of treatment protocol differences). Two other MCV-positive MCC cell lines, WaGa and MKL-2 (Figures **4D-4E** and Table S2 in File **S3**), were also re-evaluated for YM155 response *in vitro*. While our initial evaluation of WaGa *in vitro* response to YM155 is comparable to our previous data (6.0nM and 8.5nM [22], respectively), we determined with additional biological replicates that MKL-2 *in vitro* response to YM155 is more intermediate to MKL-1 and MS-1, with an EC50 value of 5.8nM (previously reported at 12.2nM [22]) (Table S3 in File **S3**). Both WaGa and MKL-2 xenografts responded *in vivo* to YM155 (4mg/kg) relative to saline treatment (P=0.0034 and P<0.0001, respectively; Table S2 in File **S3**). The comparisons of estimated mean survival on the 4mg/kg YM155 continuous treatment arm indicate that survival is prolonged greatest relative saline treatment for mice with MKL-1 xenografts, followed by MKL-2, WaGa, and finally MS-1, which do not have prolonged survival (Table S2 in File **S3**). 

### Tumor Shrinkage and Delay of Re-Growth (Regression), and/or Slower Growth Rate Is Observed upon YM155 Treatment (Relative to Saline) in Three of Four MCC Xenografts

**Figure 5:**
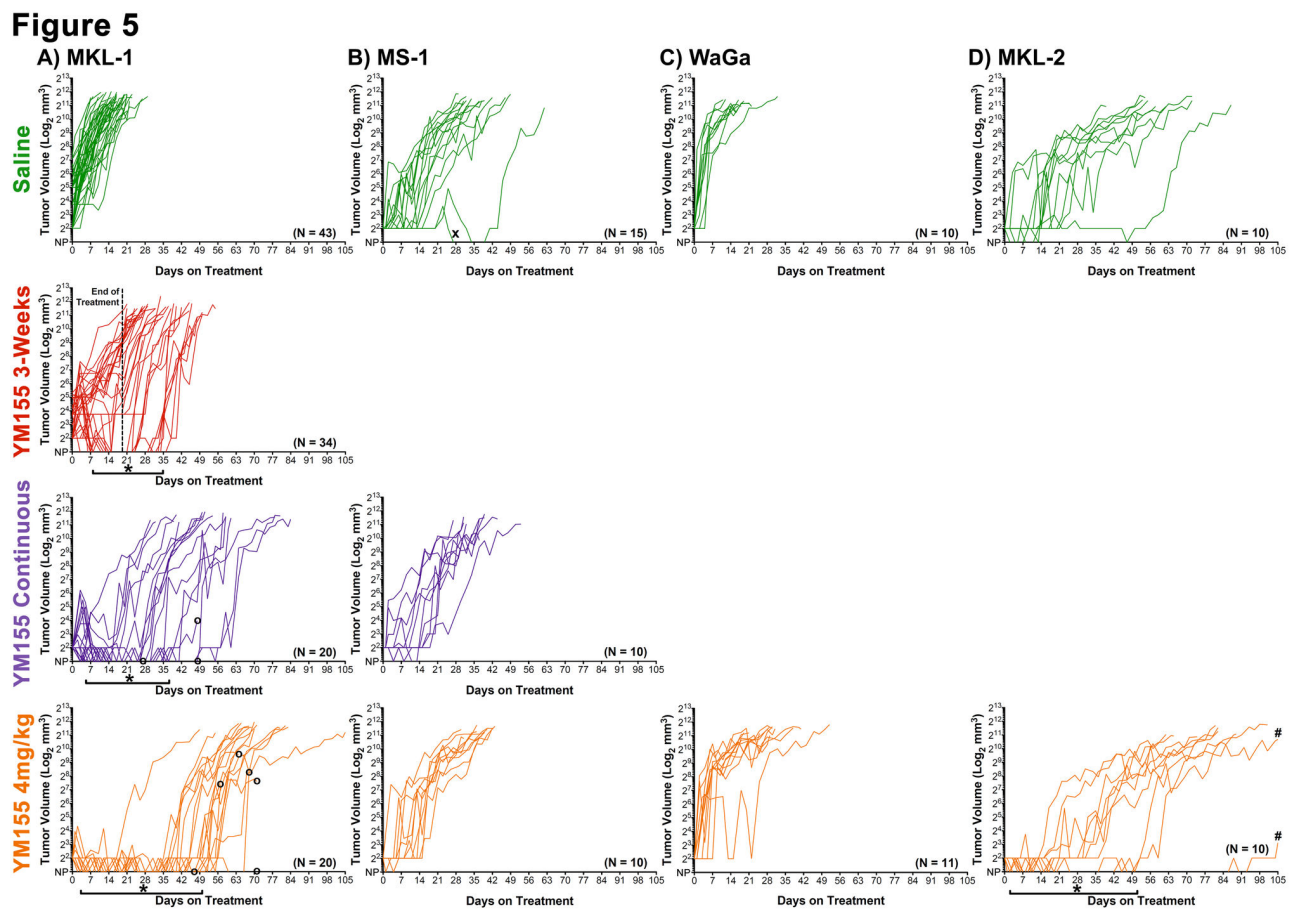
Tumor volume response to YM155 is dose, duration, and cell line dependent. Tumor volumes (mm^3^) are reported on a Log_2_ scale according to treatment group. Non-palpable (NP) tumors are indicated at baseline corresponding to tumor regression. **A**) Tumor volumes of MKL-1 xenografts undergo an initial regression period with YM155 treatment where >20% of mice lack palpable tumors (as indicated by *), which is extended with increased dose and duration of YM155 treatment. Overall tumor growth rate is reduced with increased YM155 duration and dosage. A total of 9 mice were euthanized before a diameter of 20mm was measured on the primary tumor due to distress associated with liver metastasis (as indicated by **o**). **B**) Tumor volumes of MS-1 xenografts are unaffected by YM155 treatment. A spontaneous regression was observed on saline treatment (as indicated by **x**). **C**) Tumor volumes of WaGa xenografts do not undergo an initial regression, but have a reduced growth rate. **D**) Tumor volumes of MKL-2 xenografts undergo an initial regression period with YM155 treatment where >20% of mice lack palpable tumors (as indicated by *). Overall tumor growth rate is reduced on YM155 treatment relative to saline treatment. Two mice did not reach the final 20mm tumor dimension by day 105 (as indicated by **#**).

Average tumor growth kinetics per cell line and treatment arm are reported in Table S4 in File **S3**. Tumor volume data for all 193 mice are reported in Figure **5**. Delay of tumor re-growth was significant in all YM155 treatment arms of mice with MKL-1 xenografts (Figure **5A**), relative to saline: 2mg/kg YM155 treatment for three weeks (8.6±2.5 days); 2mg/kg continuous YM155 treatment (15.8.6±3.2 days); and 4mg/kg continuous YM155 treatment (29.9±4.0 days) (Table S4 in File **S3**). The delay in re-growth was significantly greater in the 4mg/kg arm than the 2mg/kg arm (P<0.05). After the initial delay, final tumor growth rate of MKL-1 xenografts in mice treated continuously with YM155 (2mg/kg or 4mg/kg) was slower than mice treated with YM155 for 3-weeks or mice treated with saline (both P-values <0.05). Final tumor growth rates in mice treated continuously at either 2mg/kg or 4mg/kg doses were comparable (Table S4 in File **S3**). 

We next evaluated tumor growth response in MS-1 bearing mice. In our prior studies there was some noted response in tumor volume at the end of a three-week, 2mg/kg treatment period with YM155, relative to saline. However, this data corresponded to only 5 mice with no significant difference in overall survival [22]. In our current studies with increased duration of treatment and dosage, there was no shrinkage in tumor volume, delay of tumor re-growth, or reduction in growth rate observed in mice with MS-1 xenografts comparing saline treatment to YM155 treatment at either 2mg/kg or 4mg/kg (Figure **5B** and Table S4 in File **S3**). WaGa xenografts in mice treated continuously with 4mg/kg YM155 grew slower than mice treated with saline (P<0.05), but there was no evidence of initial tumor shrinkage or delay of re-growth in these mice (Figure **5C** and Table S4 in File **S3**). There was evidence of initial tumor shrinkage in YM155-treated mice with MKL-1 (Figure **5A**) and MKL-2 (Figure **5D**) xenografts (all P-values <0.05), but the absolute amount of shrinkage was small (Table S4 in File **S3**). The delay of tumor re-growth was significantly longer in mice with MKL-1 xenografts than in mice with MKL-2 xenografts (P<0.05); tumor shrinkage and delayed tumor re-growth correlate with a regression period in which >20% of mice no longer had palpable tumors (Table S4 in File **S3**, Figure **5A and 5D** **,** marked by asterisks). However, all mice were eventually euthanized due to progressive disease. Thus, while YM155 continuous treatment at 4mg/kg prolongs survival in NSG mice with three of the four MCC xenografts, this treatment regimen does not eradicate tumor cells. 

### MCV-Positive MCC Xenograft Mouse Models Develop Metastases at Different Locations in a Cell Line Dependent Manner

**Figure 6:**
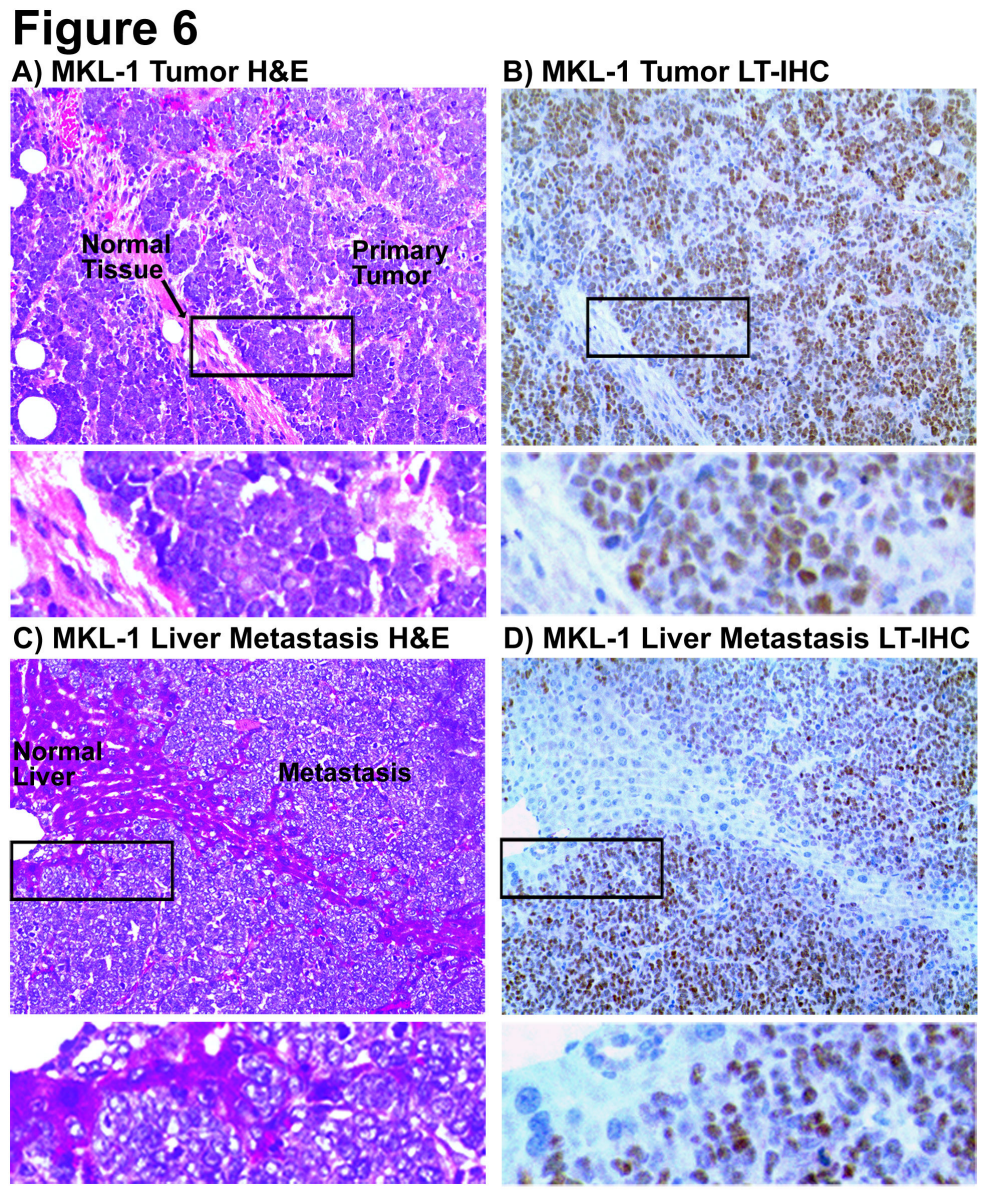
Immunohistochemistry of MCV-LT in a MKL-1 xenograft primary tumor and a liver metastasis. Shown are paired hemotoxylin & eosin (H&E) stained slides and adjacent sections stained with CM2B4, the MCV-LT antibody (LT-IHC), in mice with MKL-1 xenografts: **A**) MKL-1 xenograft primary tumor, H&E; **B**) MKL-1 xenograft primary tumor, LT-IHC; **C**) MKL-1 xenograft liver metastasis, H&E; and **D**) MKL-1 xenograft liver metastasis, LT-IHC. MKL-1 cells contains nuclear staining of LT, consistent with an intact nuclear localization signal (NLS). Original magnification = 200X; insets = 600X.

MKL-1, MS-1, and WaGa cell lines are each derived from metastatic lesions [18,42,43]; the site of MKL-2 derivation is unknown [44]. Common sites of metastasis include skin, lymph nodes, liver, lung, bone, and brain (reviewed by Eng et al [4]). Necropsy was performed on each mouse reaching experimental endpoint to assess the metastatic capability of each cell line in our mouse xenograft models. Mice with MKL-1, MS-1, or MKL-2 xenografts developed at least one or more metastases. LT-staining of primary xenograft tumors was confirmed for at least one mouse per treatment group, per cell line (data not shown). Metastatic lesions also stained positive for LT, confirming a MCC origin (Figure **6** and Figure S2 in File **S2**); 

MCC metastases occurred in the liver of 18/117 mice with MKL-1 xenografts; this subset corresponds to 27% of MKL-1-injected mice that survive past day 25. Diameters of metastatic lesions were highly variable. In 9/117 instances, liver metastasis resulted in distress requiring euthanization of mice before primary tumor diameters of 20mm were measured (Figure **5A** **,** marked by open circles). Both MKL-1 xenograft primary tumors (Figure **6A-6B**) and liver metastases (Figure **6C-6D**) contain nuclear staining for MCV-LT. Dual MKL-2 metastases occurred along the urogenital tract in 1/20 mice with separate lesion diameters of 12mm and 13mm. LT-staining in urogenital metastases was similar to staining of MKL-2 xenograft primary tumors (Figure S2A-S2D in File **S2**). MKL-2-derived MCV-LT is truncated [12,18] prior to the nuclear localization signal, or NLS [50], thus staining for LT is not restricted to the nucleus as with MKL-1 or MS-1. In one instance, a MS-1 primary tumor regressed spontaneously under saline treatment for more than 5 weeks (Figure **5B** **,** marked by x), but necropsy revealed a 3mm-diameter subcutaneous metastasis on the abdomen. This metastasis was confirmed to stain for MCV-LT, similar to MS-1 xenograft primary tumors (Figure S2E-S2H in File **S2**). Local invasion to surrounding tissues within the abdominal cavity, resulting in tumors of ~30mm diameter, was also observed in three MS-1 xenografts. WaGa xenograft primary tumors stain positive for LT (Figure S2I-S2J in File **S2**). WaGa-derived MCV-LT is truncated within the NLS [18,50], thus staining of LT is not restricted to the nucleus. WaGa-injected mice did not develop any metastases. 

### Combination Drug Treatments with YM155 Act Additively, But Not Synergistically, to Reduce MCC Cell Line Viability *In Vitro*

**Figure 7:**
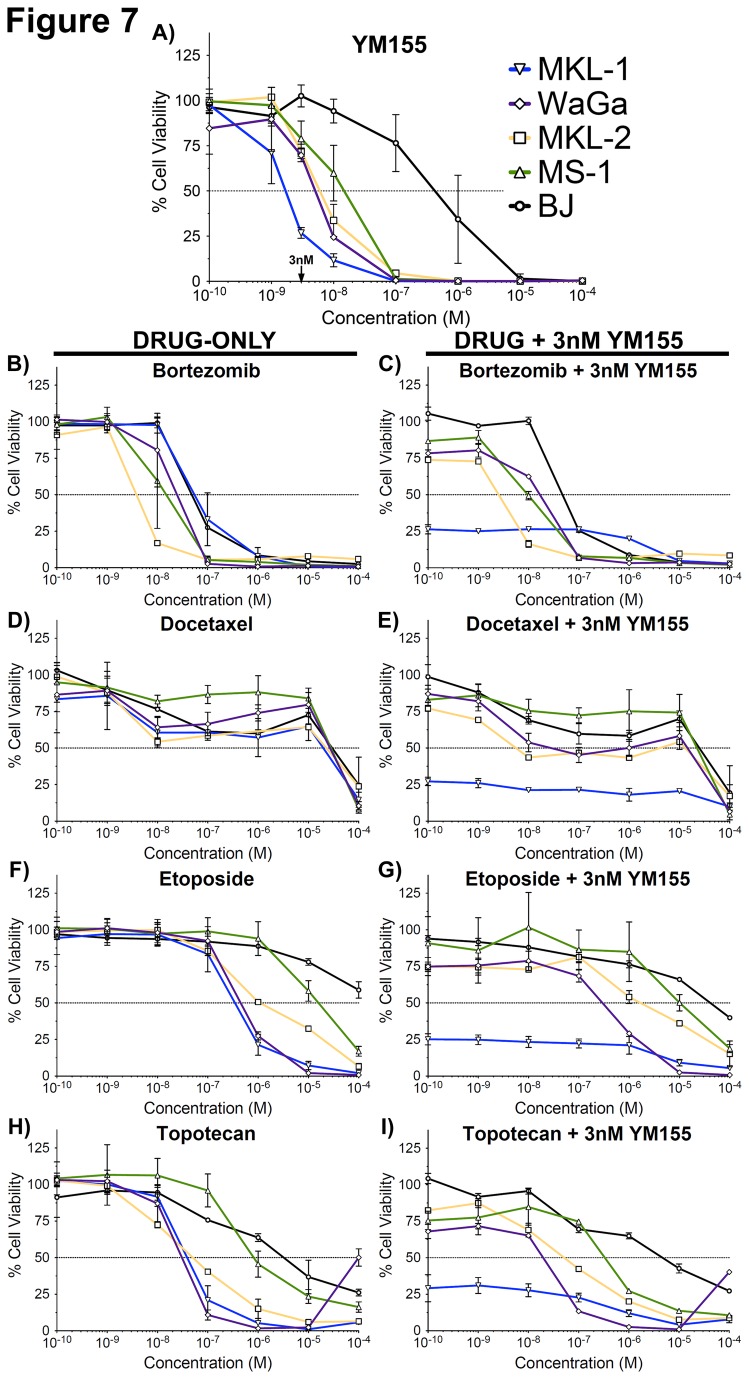
Various chemotherapeutics combined with YM155 induce MCC cell death in an additive manner, *in* *vitro*. CellTiter-GLO assays were performed using multiple MCC cell lines as well as the control primary human fibroblast, BJ. Corresponding dose-response curves are shown for the following chemotherapeutic agents and drug combinations: **A**) YM155; **B**) Bortezomib; **C**) Bortezomib + 3nM YM155; **D**) Docetaxel; **E**) Docetaxel + 3nM YM155; **F**) Etoposide; **G**) Etoposide + 3nM YM155 **H**) Topotecan; and **I**) Topotecan + 3nM YM155.

YM155 was tested alone (Figure **7A**) and in combination with other chemotherapeutic agents to identify a treatment strategy that may kill MCC cells synergistically. Bortezomib, docetaxel, carboplatin, etoposide, and topotecan were tested alone or in combination with a fixed concentration of YM155 (Figure **7B-I** **,** and Table S3 in File **S3**). Bortezomib is a proteasomal inhibitor that has been shown previously to efficiently kill MCC cells at sub-micromolar concentrations [22]. However, primary human fibroblasts, BJ, are also efficiently killed by bortezomib treatment (Figure **7B-C**). Docetaxel was previously tested in melanoma xenografts with YM155 to induce cancer-specific mitotic catastrophe and cell death [35]. Docetaxel treatment does not decrease cell viability of MCC cell lines (Figure **7D-E**). Carboplatin, a platinum-based chemotherapeutic, also does not decrease cell viability of MCC cell lines (Table S3 in File **S3**). Etoposide, a topoisomerase type II inhibitor, with or without carboplatin (data not shown), decreases cell viability of MCC cell lines at micromolar concentrations (Figure **7F-G**). Topotecan, a topoisomerase type I inhibitor, decreases cell viability at sub-micromolar concentrations (Figure **7H-I**). However, none of these chemotherapeutic agents decrease cell viability of MCC cells in a synergistic manner when combined with YM155—the effect is merely additive. EC50 values with 95% confidence intervals are reported in Table S3 in File **S3**. 

## Discussion

In this study we assessed the sensitivity of four MCV-positive MCCs to a small-molecule survivin inhibitor, YM155. Three of the four xenografts responded to YM155 treatment. YM155 efficacy is enhanced by extending the duration of treatment as well as by increasing YM155 dosage. However, the degree of YM155 efficacy is cell line dependent. Overall response to YM155 in MKL-1 xenografts, as well as a lack of overall survival to YM155 in MS-1 xenografts, is consistent with our previous observations [22]. Response to YM155 *in vivo* (Table S2 in File **S3**) reflects YM155 response *in vitro* (Figure **7A**); WaGa and MKL-2 xenografts respond to YM155 treatment intermediately compared to MKL-1 and MS-1 when assessing *in vivo* estimated survival data between YM155 4mg/kg continuous treatment and saline treatment (Table S2 in File **S3**), and they also have intermediate EC50 values determined from *in vitro* cell viability data (Figure **7A**). MKL-1 is the most sensitive to YM155 both *in vivo* and *in vitro*, whereas MS-1 is the least sensitive to YM155 *in vitro* and does not respond to YM155 *in vivo*. While relatively non-toxic, YM155 has been withdrawn from clinical development (Ann Keating, Astellas Corporation, pers. comm.); our preclinical findings suggest that survivin inhibition is a promising therapeutic approach for MCV-positive MCC. 

For MCC xenografts, regression, growth rate, and even metastatic escape are highly cell line dependent. Liver metastasis was only observed with MKL-1 xenografts, and metastasis was only observed after survival was significantly prolonged with YM155 treatment. While WaGa does not undergo regression or even tumor shrinkage upon YM155 treatment, survival was significantly prolonged relative to saline treatment owing to a reduced tumor growth rate. Why MCC xenografts stop responding to YM155 treatment and what determines overall response to YM155 for a given MCC cell line remains unknown. 

Previous studies using MCV-positive MCC cell lines identified bortezomib as a potent *in vitro* chemotherapeutic, but not *in vivo* [22]. Topoisomerase type I and type II inhibitors were also shown to induce death of MCC cell lines [22]. Although we again verified *in vitro* efficacy of bortezomib, etoposide, and topotecan, none of these agents act synergistically with YM155 treatment—the effect is only additive. However, this may not exclude the possibility that combination therapy of topoisomerase inhibitors with survivin inhibitors will prove beneficial in future studies. 

## Supporting Information

File S1 **File includes Figure S1.** Figure S1: Mouse weights prior to treatment. Mouse weights were recorded at least once weekly upon arrival and at greater intervals after cell line injection and/or upon signs of distress. Average mouse weights with standard deviations (black line) prior to treatment are reported, with the final weight record adjusted to remove the newly palpable (~2mm x 2mm) tumor volume. Maximum (red-dashed line) and minimum (blue-dashed line) mouse weights are also indicated.
(TIF)

File S2 **File includes Figure S2.** Figure S2: Immunohistochemistry of MCV-LT in MCC primary tumors and metastases. Shown are paired hemotoxylin & eosin (H&E) stained slides and adjacent sections stained with CM2B4, the MCV-LT antibody (LT-IHC), in mice with MCC xenografts: A) MKL-2 xenograft primary tumor, H&E; B) MKL-2 xenograft primary tumor, LT-IHC; C) MKL-2 xenograft urogenital metastasis, H&E; D) MKL-2 xenograft urogenital metastasis, LT-IHC; E) MS-1 xenograft primary tumor, H&E; F) MS-1 xenograft primary tumor, LT-IHC; G) MS-1 xenograft subcutaneous metastasis, H&E; H) MS-1 xenograft subcutaneous metastasis, LT-IHC; I) WaGa xenograft primary tumor, H&E; and J) WaGa xenograft primary tumor, LT-IHC. MS-1 cells contain nuclear staining of LT, consistent with an intact nuclear localization signal (NLS). Both MKL-2 and WaGa lack an intact NLS, thus LT staining is not restricted to the nucleus. Original magnification = 200X; insets = 600X.
(TIF)

File S3 **File includes Tables S1, S2, S3, and S4.** Table S1: Estimated Mean Survival Statistics. Mean estimated survival statistics were calculated for each MCC xenograft and treatment arm. C.I. = confidence interval. Table S2: Comparative Survival Statistics. Different MCC xenografts and treatment arms were cross-compared to determine differences in estimated survival. Pr = probability; ****P<0.0001; ***P<0.001; **P<0.01; *P<0.1; NS = not significant. Table S3: EC50 Values (M). MCC cell lines were evaluated for cell viability over a range of different concentrations of chemotherapeutic agents, where EC50 values are reported. C.I. = confidence interval; N.D. = not determined; N.S.C. = non-sigmoidal curve, value cannot be determined. Table S4: Average Tumor Growth Kinetics. Tumor volumes were assessed for differential growth across treatment groups using an extension to the piecewise linear hierarchical Bayesian model that accounts for batch effects. A delay in tumor growth (or re-growth) is estimated by a hinge point, called nadir, where the volume at nadir (α) is expressed as a log2(volume) and the time at nadir (ρ) is expressed in days. An initial decrease in growth is estimated as β1, where log2(volume) = α+β1*(ρ-day). Final increase in tumor growth is estimated as β2, where log2(volume) = α+β2*(day-ρ). The mean estimates and 95% confidence intervals are reported for these four parameters for each treatment and cell line. Corresponding regression periods (range, in days) where >20% of mice no longer had palpable tumors is indicated where appropriate. α = Log2 Tumor Volume at Nadir; β1 = Pre-Nadir Slope (Decreasing); β2 = Post-Nadir Slope (Increasing); ρ = Time at Nadir; Reg. = Regression; Std. Err. = Standard Error; C.I. = Confidence Interval; and N/A = Not Applicable.
(XLSX)
