# Supplementary figures and images for: Response of Merkel Cell Polyomavirus-Positive Merkel Cell Carcinoma Xenografts to a Survivin Inhibitor
Source: PLoS One. 2013 Nov 18;8(11):e80543. doi: 10.1371/journal.pone.0080543 (PMC3832378; doi:10.1371/journal.pone.0080543)

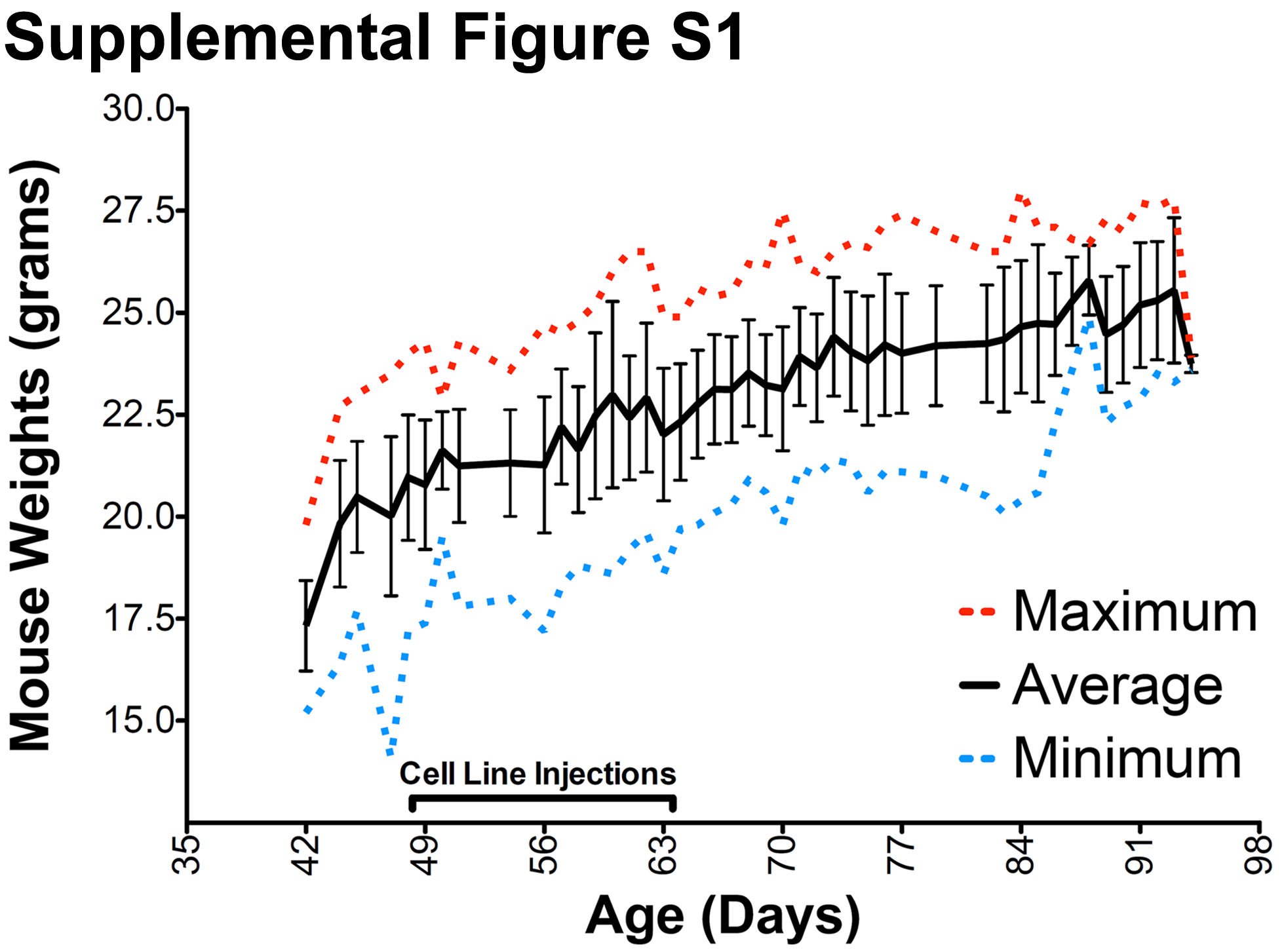

Supplement: File S1 — File includes Figure S1. Figure S1: Mouse weights prior to treatment. Mouse weights were recorded at least once weekly upon arrival and at greater intervals after cell line injection and/or upon signs of distress. Average mouse weights with standard deviations (black line) prior to treatment are reported, with the final weight record adjusted to remove the newly palpable (~2mm x 2mm) tumor volume. Maximum (red-dashed line) and minimum (blue-dashed line) mouse weights are also indicated. (TIF) [file pone.0080543.s001.tif]

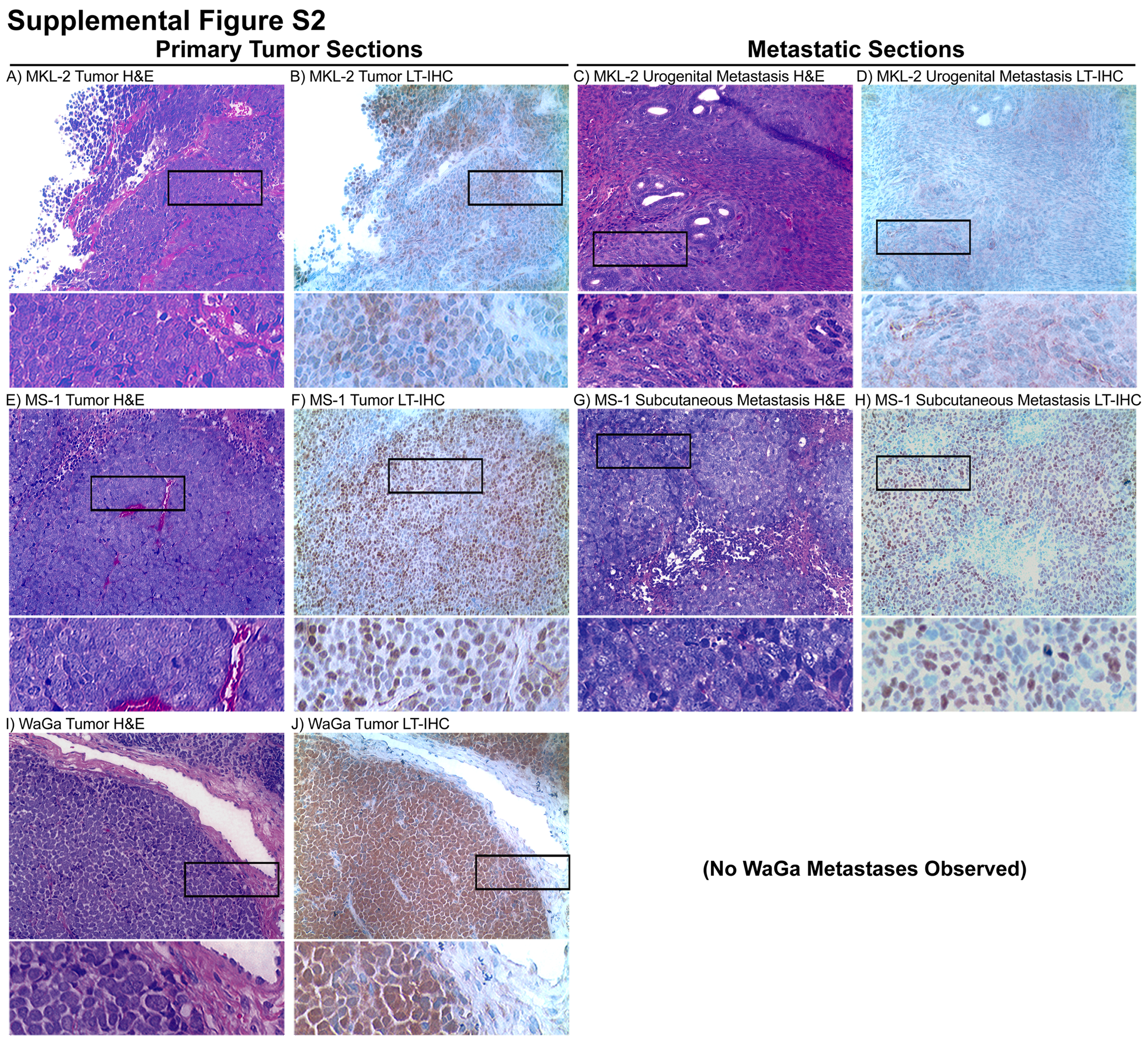

Supplement: File S2 — File includes Figure S2. Figure S2: Immunohistochemistry of MCV-LT in MCC primary tumors and metastases. Shown are paired hemotoxylin & eosin (H&E) stained slides and adjacent sections stained with CM2B4, the MCV-LT antibody (LT-IHC), in mice with MCC xenografts: A) MKL-2 xenograft primary tumor, H&E; B) MKL-2 xenograft primary tumor, LT-IHC; C) MKL-2 xenograft urogenital metastasis, H&E; D) MKL-2 xenograft urogenital metastasis, LT-IHC; E) MS-1 xenograft primary tumor, H&E; F) MS-1 xenograft primary tumor, LT-IHC; G) MS-1 xenograft subcutaneous metastasis, H&E; H) MS-1 xenograft subcutaneous metastasis, LT-IHC; I) WaGa xenograft primary tumor, H&E; and J) WaGa xenograft primary tumor, LT-IHC. MS-1 cells contain nuclear staining of LT, consistent with an intact nuclear localization signal (NLS). Both MKL-2 and WaGa lack an intact NLS, thus LT staining is not restricted to the nucleus. Original magnification = 200X; insets = 600X. (TIF) [file pone.0080543.s002.tif]
